# The relationship between changes in functional connectivity gradients and cognitive–emotional disorders in sudden sensorineural hearing loss

**DOI:** 10.1093/braincomms/fcae317

**Published:** 2024-09-19

**Authors:** Biao Li, Xiao-Min Xu, Yuan-Qing Wu, Xiu-Qian Miao, Yuan Feng, Yu-Chen Chen, Richard Salvi, Jin-Jing Xu, Jian-Wei Qi

**Affiliations:** Department of Otolaryngology, Nanjing First Hospital, Nanjing Medical University, Nanjing 210006, China; Department of Radiology, Nanjing First Hospital, Nanjing Medical University, Nanjing 210006, China; Department of Otolaryngology, Nanjing First Hospital, Nanjing Medical University, Nanjing 210006, China; Department of Radiology, Nanjing First Hospital, Nanjing Medical University, Nanjing 210006, China; Department of Radiology, Nanjing First Hospital, Nanjing Medical University, Nanjing 210006, China; Department of Radiology, Nanjing First Hospital, Nanjing Medical University, Nanjing 210006, China; Center for Hearing and Deafness, University at Buffalo, The State University of New York, Buffalo, NY 14214, USA; Department of Otolaryngology, Nanjing First Hospital, Nanjing Medical University, Nanjing 210006, China; Department of Otolaryngology, Nanjing First Hospital, Nanjing Medical University, Nanjing 210006, China

**Keywords:** sudden sensorineural hearing loss, functional magnetic resonance imaging, functional connectivity gradients, cognitive–emotional disorders

## Abstract

Sudden sensorineural hearing loss, a prevalent emergency in otolaryngology, is known to potentially precipitate cognitive and emotional disorders in affected individuals. Extensive research has documented the phenomenon of cortical functional reorganization in patients with sudden sensorineural hearing loss. However, the potential link between this neural functional remodelling and cognitive–emotional disorders remains unclear. To investigate this issue, 30 bilateral sudden sensorineural hearing loss patients and 30 healthy adults were recruited for this study. We collected clinical data and resting-state functional magnetic resonance imaging data from the participants. Gradient mapping analysis was employed to calculate the first three gradients for each subject. Subsequently, gradient changes in sudden sensorineural hearing loss patients were compared with healthy controls at global, regional and network levels. Finally, we explored the relationship between gradient values and clinical variables. The results revealed that at the global level, sudden sensorineural hearing loss did not exhibit significant differences in the primary gradient but showed a state of compression in the second and third gradients. At the regional level, sudden sensorineural hearing loss patients exhibited a significant reduction in the primary gradient values in the temporal pole and ventral prefrontal cortex, which were closely related to neuro-scale scores. Regarding the network level, sudden sensorineural hearing loss did not show significant differences in the primary gradient but instead displayed significant changes in the control network and default mode network in the second and third gradients. This study revealed disruptions in the functional hierarchy of sudden sensorineural hearing loss, and the alterations in functional connectivity gradients were closely associated with cognitive and emotional disturbances in patients. These findings provide new evidence for understanding the functional remodelling that occurs in sudden sensorineural hearing loss.

## Teaser

Li *et al*. reported that patients with bilateral sudden sensorineural hearing loss experience remodelling of the cerebral cortex’s connectivity gradients, which correlates with their cognitive and emotional disorders. The loss of sensory functions leads to abnormalities in higher-order cognitive networks.

## Graphical Abstract

**Graphical Abstract:**
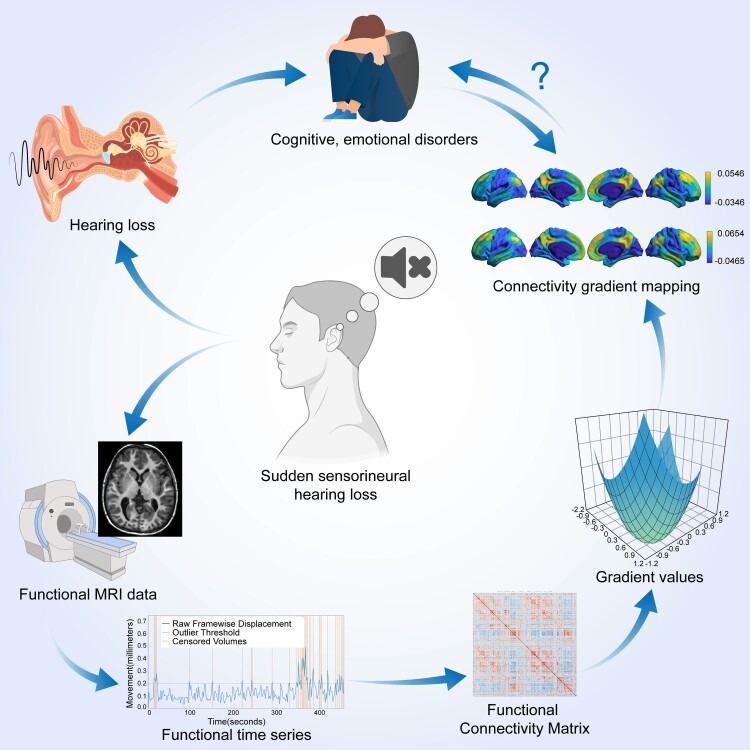

## Introduction

Sudden sensorineural hearing loss (SSNHL) is defined as an unexplained rapid decline in hearing over 3 days, features of at least 20 dB at two adjacent frequencies. It is a common type of sensorineural hearing loss.^1^ In addition to the primary symptom of hearing loss, patients often experience tinnitus, vertigo or a feeling of fullness in the ears, significantly increasing their discomfort. However, the aetiology of SSNHL remains unclear, with possible causes including viral infections, vascular diseases, autoimmune disorders and tumours. The incidence of SSNHL in Western populations is approximately five in 100 000 individuals, with bilateral SSNHL accounting for only 0.4–3.4% of all cases.^2,3^ The incidence of SSNHL is rising and tends to affect younger individuals.^4^ Although interventions like corticosteroid injections can alleviate or even cure SSNHL to some extent, over two-thirds of patients do not fully recover. Prolonged hearing loss and its accompanying symptoms significantly reduce patients’ quality of life.

Research has also shown that the risk of concurrent depression in SSNHL patients is 1.29 times higher than in the healthy population, and the risk of SSNHL in patients with depression is 2.17 times higher than in normal individuals, a phenomenon also confirmed by another study.^5-7^ Additionally, the risk of concurrent anxiety disorders is higher in SSNHL patients.^7^ Past research and clinical manifestations indicate that the likelihood of emotional disorders in SNNHL patients is increased, and the occurrence of these disorders may exacerbate SSNHL or affect its prognosis, forming a vicious cycle. Neuroimaging studies corroborate alterations in higher-order cognitive networks, such as the default mode network (DMN) and the salience network (SN), due to auditory impairment.^8-10^ Such disruptions in advanced information processing may adversely impact speech comprehension, working memory and executive functions.^11-13^ Despite copious evidence illustrating cortical functional reorganization resulting from SSNHL,^14-17^ However, previous studies have largely focused on unilateral SSNHL. Research has demonstrated that compared to unilateral SSNHL, patients with bilateral SSNHL exhibit a more severe decline in hearing and poorer clinical prognoses.^18^ Moreover, the changes in brain network functionality in patients with bilateral SSNHL and its precise relationship with cognitive–emotional disorders remain elusive.

Past research has primarily focused on functional connectivity changes between specific networks, neglecting the alterations in brain regions outside these networks. Consequently, these studies failed to comprehensively demonstrate the alterations in cortical functional connectivity in SSNHL. The introduction of cortical gradient theory has changed this situation. Studies have confirmed the existence of microstructural gradients in the human brain cortex, radiating from sensory-motor areas to higher-order regions in the parietal, temporal and frontal cortices.^19-21^ As research progresses, the focus has shifted to the study of macroscopic connectome gradients. Utilizing diffusion embedding techniques, it has been demonstrated that in a healthy adult brain, cortical network connectivity gradients are linearly distributed, with unimodal sensory networks like visual and somatosensory at one end, and higher-order cognitive networks like the default mode and limbic networks at the other.^22^ This functional connectivity gradient aligns with previous microstructural cortical gradients, suggesting a close relationship between macroscopic connectome gradients and local microstructural gradients, indicating that cortical functional connectivity has spatial and hierarchical structures.^23^ This analysis method makes the spatial changes in cortical functional connectivity more tangible. Compared to common functional connectivity analysis methods (which divide the cortex into distinct regions for point-to-point connectivity analysis), gradient functional connectivity mapping can reflect the spatial continuity of changes in cortical functional connectivity.^24^ Gradient mapping methods use decomposition or embedding techniques to identify the principal axes of variance in data, replacing the original dimensions with a new set of dimensions. After several replacements, each cortical location can be described by a set of values reflecting its position in the new dimensions. This allows for the identification of brain functional hierarchies in a continuous low-dimensional space,^24^ overcoming the limitations of traditional analysis methods in macroscopically describing brain functional connectivity and reflecting its spatial continuity.

Previous scholars have used connectome gradient technology to confirm changes in the primary gradient in patients with severe depression, both globally and locally.^25^ Studies on other psychiatric disorders have also reported disruptions in functional hierarchies.^26,27^ This leads us to speculate whether SSNHL bilateral patients also exhibit topological reorganization of functional connectivity.

We hypothesize that: (i) bilateral SSNHL patients exhibit disorganized whole-brain cortical connection gradients; and (ii) regions where functional connectivity gradients change are linked to cognitive and emotion performance. In this study, we aimed to test our hypothesis by collecting functional magnetic resonance imaging (fMRI) data in both bilateral SSNHL patients and a healthy control group, to compute and compare the functional connectivity gradients across global, local and network levels. We also explored their correlation with cognitive and emotion performance.

## Materials and methods

### Subjects and clinical assessment

The study included a total of 30 patients with bilateral SSNHL and 30 healthy control (HC) individuals matched for age, gender and education level. Participants were recruited from local communities and otolaryngology departments in hospitals. Prior to participating in the study, informed consent was obtained from all subjects.

Inclusion criteria for SSNHL patients were as follows: (i) age range of 18–65 years; (ii) right-handedness; (iii) education level of at least 9 years; (iv) a decrease in hearing thresholds of at least 20 dB in at least three adjacent frequencies in both ears; and (v) duration of symptoms < 2 weeks. Exclusion criteria were as follows: (i) history of Ménière’s disease, auditory hypersensitivity, pulsatile tinnitus or ear surgery; (ii) history of clinical stroke or traumatic brain injury; (iii) previous central nervous system disorders, psychiatric disorders or family history of dementia; (iv) severe cardiovascular, hepatic or renal diseases, chronic debilitating diseases, hyperthyroidism or hypothyroidism; (v) history of alcohol and/or substance dependence, use of cognitive-enhancing, antidepressant or antipsychotic medications; and (vi) contraindications for MRI scanning.

All procedures in our study were in accordance with The Code of Ethics of the World Medical Association (Declaration of Helsinki) for experiments involving humans and approved by the Research Ethics Committee of the Nanjing Medical University.

### Neuropsychological assessment

The subjects underwent a battery of psycho-psychological assessments, including the Mini-Mental State Exam (MMSE), which is commonly used to screen for cognitive decline.^28^ The Self-rating Anxiety Scale (SAS) and Hamilton Depression Scale were employed to evaluate the mental and psychological status, specifically anxiety and depression, of the participants.^29^ To assess learning and verbal memory, the Auditory Word Learning Test (AVLT) and AVLT-delay were administered.^30^ Additionally, the Symbol Digit Modalities Test (SDMT) was utilized to evaluate the information processing speed of the participants.^31^

### MRI data acquisition

Imaging data were acquired from all participants using an eight-channel head coil on a 3.0 Tesla Philips MRI scanner. During the MRI scan, subjects were instructed to remain still, clear their minds, stay awake and avoid thinking about anything specific. Resting-state functional magnetic resonance imaging (rs-fMRI) scans were performed for a duration of 8 min and 8 s using gradient echo planar imaging (EPI) sequences. The imaging parameters were as follows: echo time (TE) = 30 ms, 36 slices with a thickness of 4 mm, repeat time (TR) = 2000ms, no gap between slices, field of view (FOV) = 240 mm × 240 mm, matrix size = 64 × 64, flip angle (FA) = 90° and voxel size = 3.75 mm × 3.75 mm × 4.0 mm. Additionally, three-dimensional turbine fast echo (3D-TFE) T_1_-weighted (T1WI) sequences were used to obtain structural images. The parameters for the T1WI sequence were as follows: field of view (FOV) = 256 mm × 256 mm, TR/TE = 8.1/3.7 ms, 170 slices with a thickness of 1 mm, no gap between slices, matrix size = 256 × 256, and flip angle (FA) = 8°. The acquisition time for the T1WI sequence was 5 min and 29 s. All scans were performed using parallel imaging with sensitivity encoding (SENSE) technology, with a SENSE factor of 2.

### Processing

We employed the fMRI Prep pipeline for preprocessing of fMRI data.^32^ For structural data, intensity inhomogeneity correction and skull stripping were performed using normalization tools, and these data were used as T_1_-weighted (T1WI) references for functional data processing. The boundary-based registration algorithm in FreeSurfer was utilized to align each subject’s structural and functional data. Subsequently, motion correction was carried out using FSL. Considering the temporal differences in data acquisition, slice timing correction was applied to the functional data of each subject, and their BOLD time series were transformed back into the standard MNI space. This process included head motion correction and susceptibility distortion correction. Based on the T1w reference, global signals from three regions—CSF, white matter (WM) and grey matter—were extracted from the BOLD time series using FSL. The processed data in the standard space were further denoised using 36 regression variables in xcpEngine, including six types of head motion measurements, overall signal, average WM signal, average CSF signal, their temporal derivatives and quadratic expansions.^33^ The data were then filtered using a temporal filter ranging from 0.01 to 0.08 Hz. Finally, the preprocessed functional data were projected onto the cortical surface using FreeSurfer’s mri_vol2surf, and the surface BOLD time series were smoothed with a 6 mm smoothing kernel.

### Connectome gradient analysis

Upon preprocessing, the average time series of 400 regions on the cortical surface were extracted. Subsequently, a functional connectivity matrix of size 400 × 400 was constructed for each participant based on Pearson correlation^34^ (refer to the code in the Supplementary file: step_0_convert_xcp_pconn_fisher_z.m). BrainSpace toolbox was employed for gradient mapping. Following this, cosine similarity between nodes was computed using Pearson correlation, resulting in a final similar functional connectivity matrix. The diffusion map embedding algorithm was then applied to the data to identify gradient components explaining functional connectivity variance (refer to the code in the Supplementary file: step_1_all_subjects_gradients.m). Iterative Procrustes rotation was performed to align the gradient maps across individuals (refer to code in the supplementary file: step_2_align_all_subjects.m). Evaluation was conducted on the connectivity variance of the first 19 gradient components (λ-values). The focus of this experiment lies on the first three gradients, with computation of gradient explanation ratio, range and variability as global-level gradient metrics. Using age and gender as covariates, a general linear model (GLM) was employed to assess the inter-group differences in the aforementioned indicators at the first gradient (refer to code in the Supplementary file: step_3_group_stats.m).

### Network-level gradient analysis

In order to compare gradients between networks, the 400 regions of interest (ROIs) were divided into seven networks: the DMN, control network (CON), limbic network (LIM), salience attention network (SAL), dorsal attention network (DAN), somatomotor network (SOM) and visual network (VIS).^35^ The average gradient values for each network were calculated, and independent sample *t*-tests were performed using SPSS software to determine the inter-group differences in gradients across various networks, with a significance level set at *P* < 0.05.

### Local gradient analysis

To identify the changes in functional connectivity gradients at the regional level, we utilized the GLM, with age and gender as covariates, to compare the gradient values of each voxel between the SSNHL and HC groups. The results were corrected for false discovery rate (FDR), and the significance threshold was set at *P* < 0.05.

### Statistical analysis of demographic and clinical variables

For the demographic and clinical data of the two groups, we conducted the analysis using SPSS version 26.0. For data that followed a normal distribution, paired *t*-tests were employed. For data that did not follow a normal distribution, analysis was performed using variance analysis (ANOVA) or non-parametric tests as appropriate. A significance level was set at *P* < 0.05.

### Correlation analysis of altered connectome gradient values with clinical variables

To analyse the correlation between functional connectivity gradients and neuro-scale scores, we selected brain regions that exhibited significant inter-group differences as our targets. We then extracted their gradient values and performed linear regression analysis with the neuro-scale scores.

## Result

### Demographic and clinical characteristics

The demographic data and clinical characteristics of the participants are presented in Table 1. There were no significant differences between the two groups in terms of gender, age or educational level. However, significant inter-group differences were observed in all hearing frequencies (*P* < 0.001). In this study, all SSNHL patients exhibited varying degrees of hearing loss, ranging from mild to severe. Regarding the neuro-scale scores, except for MMSE, all other scores showed significant inter-group differences (*P* < 0.05).

**Table 1:** Demographic information and clinical characteristics

	SSNHL	HCs	*P*-value
Demographic information			
Number of subjects	30	30	
Gender (Male/Female)	16/14	17/13	0.129
Age (years)	53.40 ± 8.57	50.23 ± 7.36	0.130
Education (years)	11.40 ± 2.91	13.10 ± 4.06	0.067
Duration	11.57 ± 4.86		
Neuropsychological tests			
MMSE	29.90 ± 0.31	30.00 ± 0.00	0.083
SAS	43.67 ± 7.81	33.37 ± 4.23	<0.001***
HAMD	8.00 ± 4.21	4.63 ± 1.59	<0.001***
SDMT	31.47 ± 9.16	46.77 ± 10.31	<0.001***
AVLT	3.77 ± 1.33	4.60 ± 1.38	0.021*
AVLT_delay	4.83 ± 2.04	7.20 ± 1.83	<0.001***
Hearing thresholds of each ear			
Right—0.25 kHz	53.00 ± 33.10	22.50 ± 5.37	<0.001***
Right—0.5 kHz	54.33 ± 34.78	16.50 ± 4.76	<0.001***
Right—1 kHz	57.17 ± 34.46	16.50 ± 3.97	<0.001***
Right—2 kHz	66.50 ± 23.49	14.33 ± 4.87	<0.001***
Right—4 kHz	66.77 ± 22.69	17.00 ± 6.10	<0.001***
Right—8 kHz	66.00 ± 24.12	16.33 ± 5.86	<0.001***
Left—0.25 kHz	49.33 ± 20.87	22.67 ± 5.37	<0.001***
Left—0.5 kHz	45.67 ± 24.17	18.67 ± 6.15	<0.001***
Left—1 kHz	51.83 ± 25.75	17.83 ± 3.87	<0.001***
Left—2 kHz	52.83 ± 23.37	16.17 ± 6.65	<0.001***
Left—4 kHz	55.93 ± 20.75	19.33 ± 6.26	<0.001***
Left—8 kHz	60.17 ± 22.95	16.33 ± 5.71	<0.001***

Data are expressed as mean ± standard deviation. *P*-values were calculated with the independent *t*-test or χ^2^ test, as appropriate. SSNHL, sudden sensorineural hearing loss; HCs, healthy controls; MMSE, Mini-Mental State Exam; SAS, Self-rating Anxiety Scale; HAMD, Hamilton Depression Scale; SDMT, Symbol Digit Modalities Test; AVLT, Auditory Verbal Learning Test.

^*^ *P* < 0.05, ***P < 0.001.

### The characteristics of the first three gradients

**Figure 1:**
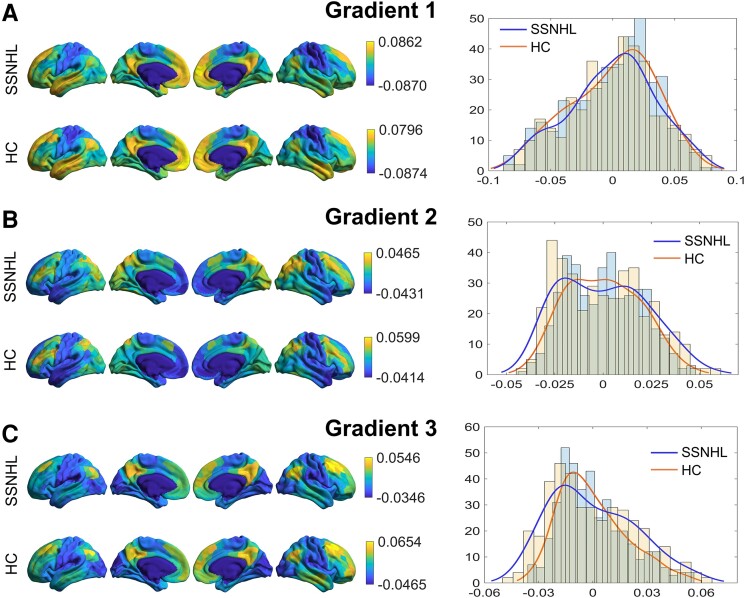
**Connectome gradient mapping for the SSNHL and HC groups.** (**A**) The distribution of individual-averaged principal gradient scores on the cortical surface in individuals with SSNHL and HC. The global histogram of principal gradient related to the 400 regions in individuals with SSNHL and HC. (**B**) The distribution of individual-averaged secondary gradient scores on the cortical surface in individuals with SSNHL and HC. The global histogram of the secondary gradient related to the 400 regions in individuals with SSNHL and HC. (**C**) The distribution of individual-averaged third gradient scores on the cortical surface in individuals with SSNHL and HC. The global histogram of the third gradient related to the 400 regions in individuals with SSNHL and HC. SSNHL, sudden sensorineural hearing loss; HC, healthy control.

**Figure 2:**
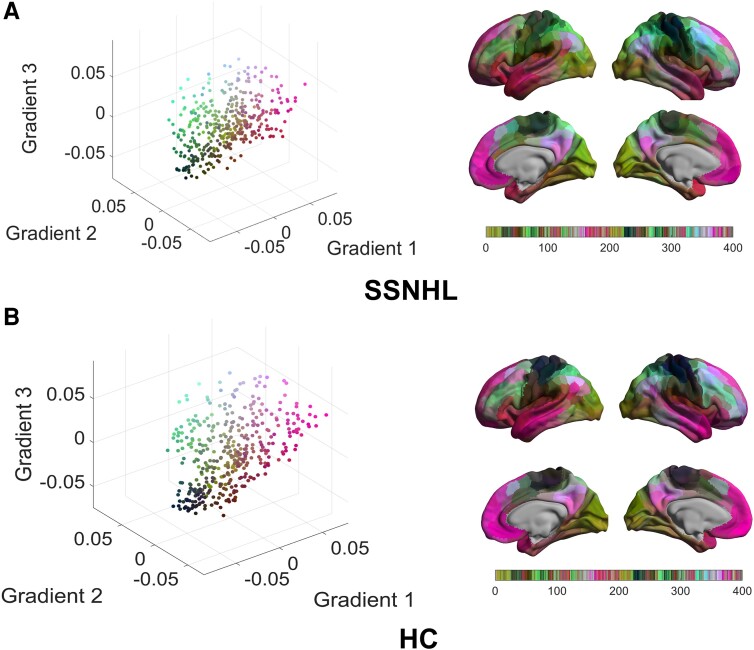
**The scatter plot of the first three gradient scores in the SSNHL and HC groups.** (**A**) The scatter plot of the first three gradient scores in patients with SSNHL. (**B**) The scatter plot of the first three gradient scores in patients with HC. SSNHL, sudden sensorineural hearing loss; HC, healthy control.

The first three gradients collectively explained 41% of the variance, with the first gradient accounting for 20%, the second gradient for 12% and the third gradient for 9%.^36^ The connectome gradient mappings for the SSNHL and HC groups were shown in Fig. 1. On the primary gradient, both groups exhibited a gradient distribution pattern from the SOM to the DMN (Fig. 1A). The second gradient demonstrated a separation between the SOM and the visual network, consistent with previous research findings (Fig. 1B). In the third gradient, the participants exhibited a distribution pattern from the VIS/SOM to the CON/DMN (Fig. 1C). Furthermore, the second and third gradients both exhibited a state of compression (Fig. 1B and C). Figure 2 presents a scatter plot of the first three gradients for both groups of SSNHL (Fig. 2A) and HC (Fig. 2B).

### The characteristics of the primary gradient

**Figure 3:**
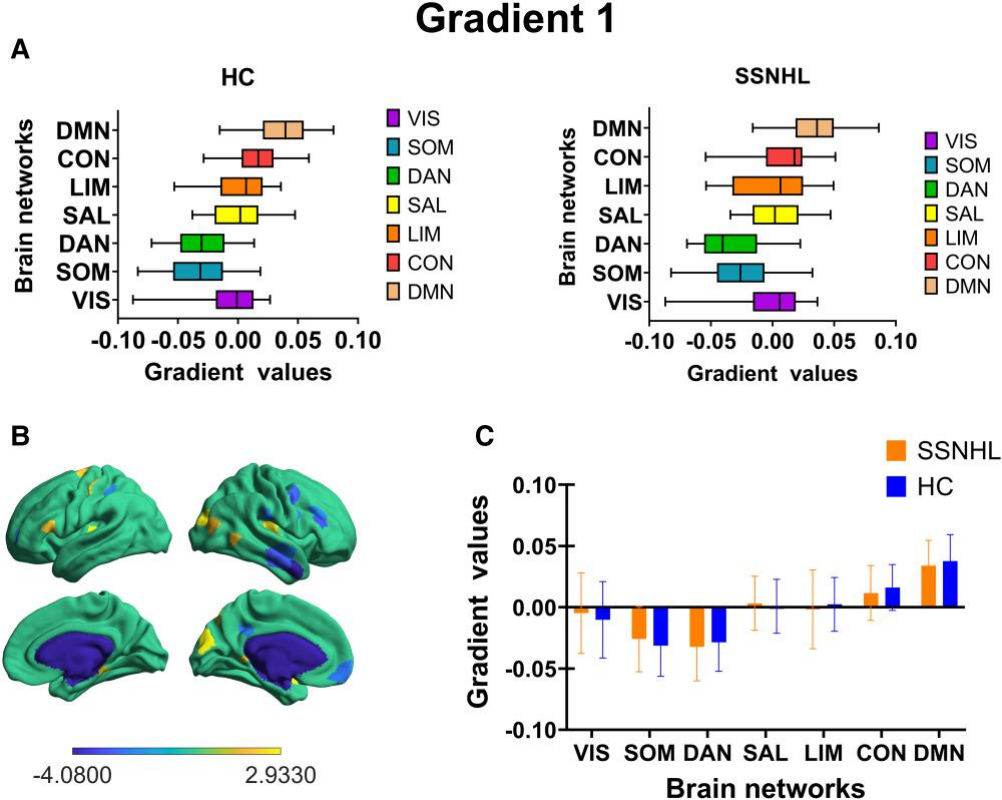
**The functional network gradient comparisons in principal gradient between SSNHL (*n* = 30) and HC groups (*n* = 30) assessed by two-sample *t*-test.** (**A**) First gradient values distribution in seven functional networks. (**B**) The region-level differences of the principal gradient between two groups (two-sample *t*-test, *P* < 0.05, FDR uncorrected). The heat map scale bar displays the range of *t*-values for distinct brain regions, where the magnitude of these values reflects the degree of between-group differences in each region. Larger absolute values indicate greater between-group differences in that brain region. (**C**) The network-level differences of the principal gradient between two groups. Through the two-sample *t*-test, no significant group differences were observed at the network level (two-sample *t*-test, *P* = 0.05, FDR uncorrected). SSNHL, sudden sensorineural hearing loss; HCs, healthy controls; DMN, default mode network; CON, control; LIM, limbic; SAL, salience attention; DAN, dorsal attention; SOM, somatomotor; VIS, visual; TP, temporal pole; vPFC, ventral prefrontal cortex.

**Figure 4:**
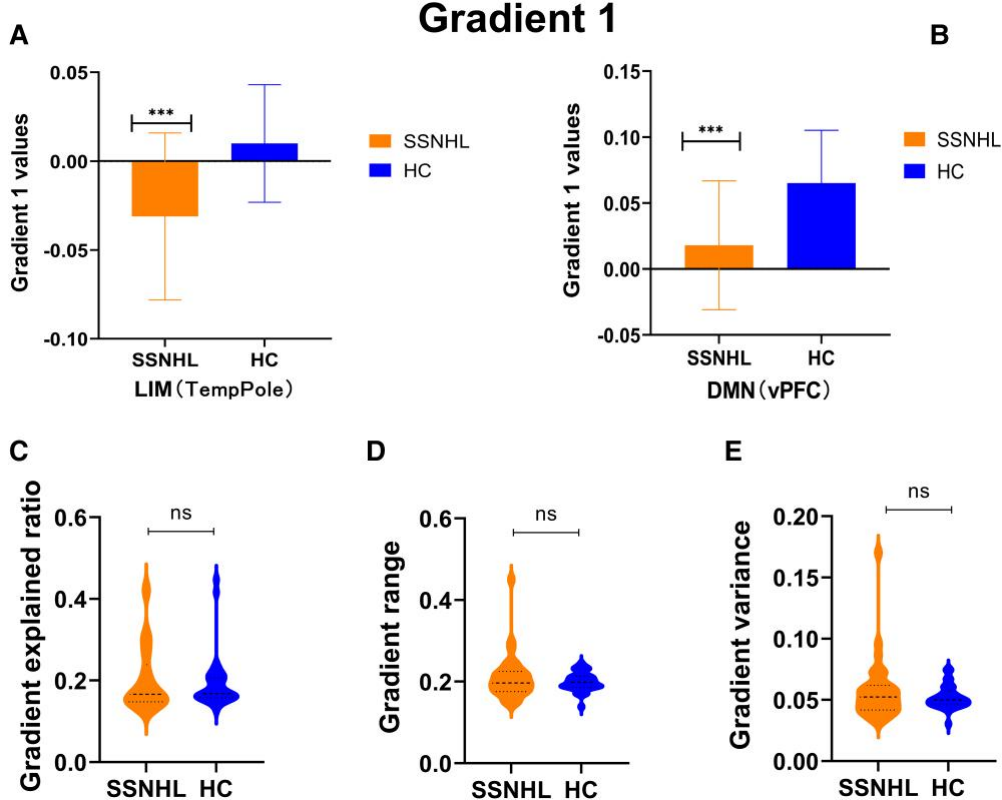
**The local gradient differences in principal gradient between SSNHL (*n* = 30) and HC groups (*n* = 30) assessed by two-sample *t*-test.** (**A**). Group differences in the TP temporal pole that had significance differences (two-sample *t*-test, FDR-corrected *P* < 0.05). In the TP temporal pole, the gradient values in SSNHL were significantly lower than those in HC. (**B**) Group differences in the vPFC that had significance differences (two-sample *t*-test, FDR-corrected *P* < 0.05). In the vPFC, the gradient values in SSNHL were significantly lower than those in HC. (**C**) The differences of the gradient range between patients with SSNHL and HC (two-sample *t*-test, *P* > 0.05). (**D**) The differences of the gradient variance between patients with SSNHL and HC (two-sample *t*-test, *P* > 0.05). (**E**) The differences of the gradient explained ratio between patients with SSNHL and HC (two-sample *t*-test, *P* > 0.05). SSNHL, sudden sensorineural hearing loss; HCs, healthy controls; DMN, default mode network; CON, control; LIM, limbic; SAL, salience attention; DAN, dorsal attention; SOM, somatomotor; VIS, visual; TP, temporal pole; vPFC, ventral prefrontal cortex. ***P < 0.005; ns, no significant.

On the primary gradient, both groups exhibited a gradient distribution pattern from the SOM to the DMN (Fig. 3A). In addition, a similar pattern of network gradient distribution was exhibited in both groups, without significant inter-group differences (Fig. 3C). However, at the regional level, significant inter-group differences (*P* < 0.05, after FDR correction) were observed in the temporal pole within the limbic system and the ventral prefrontal cortex (vPFC) in the DMN (Fig. 4A and B). Furthermore, on the primary gradient, there were no significant differences between the two groups in terms of gradient explained ratio, gradient range and gradient variance (Fig. 4C–E).

### The characteristics of the second gradient

**Figure 5:**
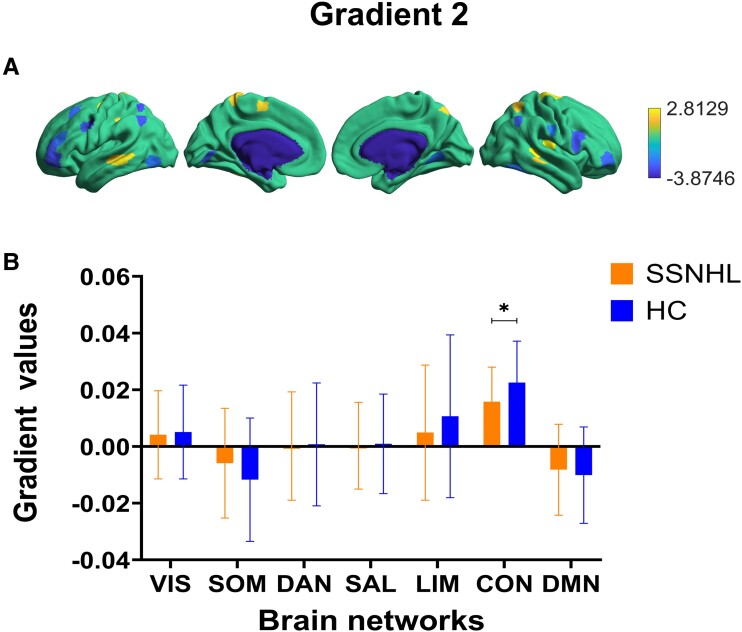
**The gradient comparisons in the second gradient between SSNHL (*n* = 30) and HC groups assessed by independent samples *t*-tests.** (**A**) The region-level differences of the principal gradient between two groups (two-sample *t*-test, *P* < 0.05, FDR uncorrected). The heat map scale bar displays the range of *t*-values for distinct brain regions, where the magnitude of these values reflects the degree of between-group differences in each region. Larger absolute values indicate greater between-group differences in that brain region. (**B**) Group differences in the second gradient values at the network level (two-sample *t*-test, *FDR-uncorrected *P* < 0.05). SSNHL, sudden sensorineural hearing loss; HC, healthy control.

At the regional level, there were also inter-group differences (*P* < 0.05, FDR uncorrected) (Fig. 5A). In the second, both groups demonstrated a separation between the SOM and the VIS. It was noting that there were significant differences in CON between the two groups (*P* < 0.05, without FDR correction) (Fig. 5B).

### The characteristics of the third gradient

**Figure 6:**
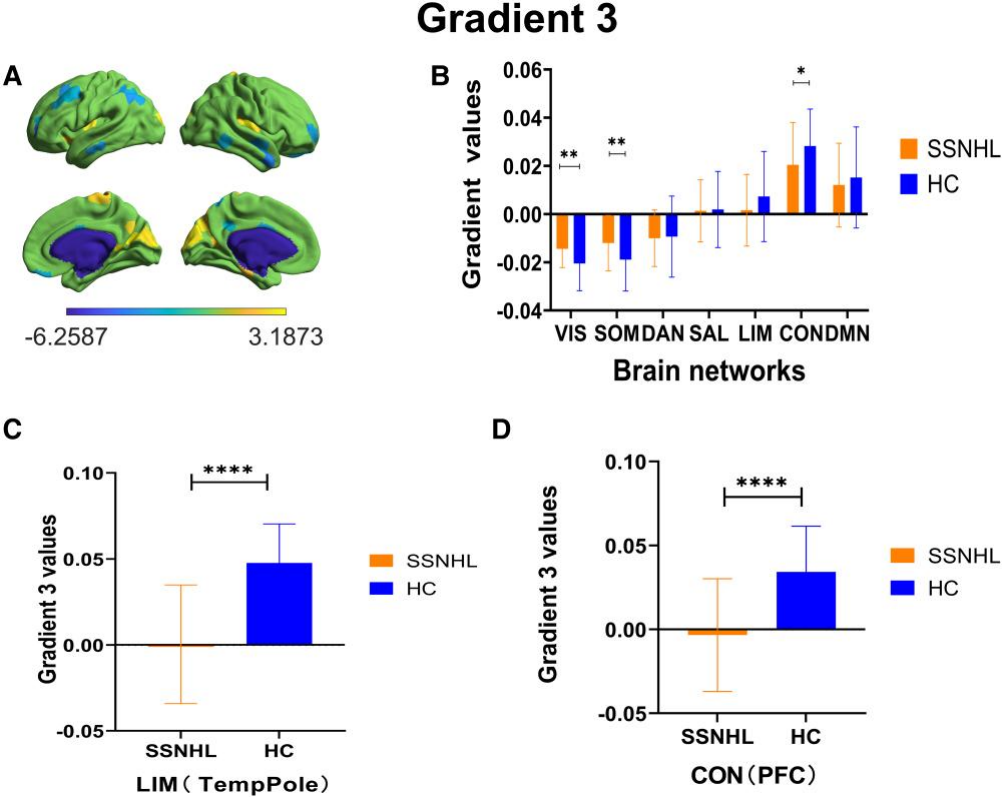
**The gradient comparisons in the third gradient between SSNHL (*n* = 30) and HC groups (*n* = 30) assessed by independent samples *t*-tests.** (**A**) In the third gradient, the region-level differences of the principal gradient between two groups (two-sample *t*-test, *P* < 0.05, FDR uncorrected). The heat map scale bar displays the range of *t*-values for distinct brain regions, where the magnitude of these values reflects the degree of between-group differences in each region. Larger absolute values indicate greater between-group differences in that brain region. (**B**) Group differences in the second gradient values at the network level (two-sample *t*-test, FDR-uncorrected *P* < 0.05). Significant inter-group differences were observed in CON, VIS and SOM between the two groups. (**C**) Group differences in the TP that had significance differences (two-sample *t*-test, FDR-corrected *P* < 0.05). (**D**) Group differences in the PFC that had significance differences (two-sample *t*-test, FDR-corrected *P* < 0.05). TP, temporal pole; vPFC, ventral prefrontal cortex; CON, control; VIS, visual; SOM, somatomotor. *P < 0.05; **P < 0.01; ****P < 0.001.

In the third gradient, the participants exhibited a distribution pattern from the VIS/SOM to the CON/DMN (Fig. 6B). Significant inter-group differences were observed in CON, VIS and SOM between the two groups. Furthermore, there were significant inter-group differences (*P* < 0.05, after FDR correction) in the temporal pole within the limbic system and the lateral prefrontal cortex in the CON (Fig. 6C and D).

### The correlation between gradient values and clinical data

**Figure 7:**
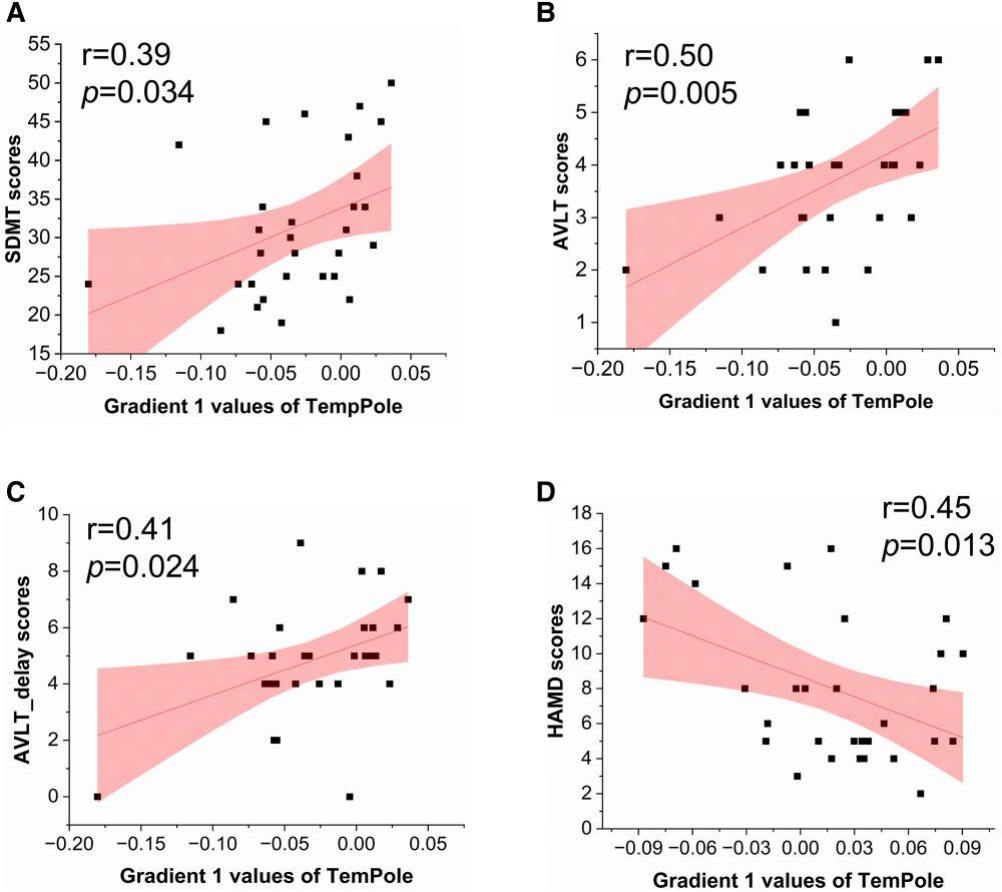
**Correlation between gradient values of ROIs and scale scores with linear regression analysis.** (**A**) The primary gradient value of the TP in SSNHL patients (*n* = 30) showed a significant positive correlation with the SDMT scores (*P* = 0.034, *r* = 0.39). (**B**) The primary gradient value of the TP in SSNHL patients (*n* = 30) showed a significant positive correlation with the AVLT scores (*P* = 0.005, *r* = 0.50). (**C**) The primary gradient value of the TP in SSNHL patients (*n* = 30) showed a significant positive correlation with the AVLT_delay scores (*P* = 0.024, *r* = 0.41). (**D**) The primary gradient value of the vPFC in SSNHL patients (*n* = 30) showed a significant negative correlation with the HAMD scores (*P* = 0.013, *r* = −0.45). ROIs, regions of interest; TP, temporal pole; SSNHL, sudden sensorineural hearing loss; SDMT, Symbol Digit Modalities Test; AVLT, Auditory Verbal Learning Test; vPFC, ventral prefrontal cortex; HAMD, Hamilton Depression Scale.

We found that in SSNHL patients, the primary gradient value in the temporal pole showed a significant positive correlation with SDMT (*r* = 0.39, *P* = 0.034), AVLT (*r* = 0.50, *P* = 0.05) and AVLT_delay (*r* = 0.41, *P* = 0.024) (Fig. 7A–C). Additionally, the primary gradient value in the prefrontal cortex exhibited a significant negative correlation with HAMD (*r* = −0.45, *P* = 0.013) (Fig. 7D).

## Discussion

In this study, we employed functional connectivity gradient analysis to assess changes in the functional connectivity gradients of patients with SSNHL at global, regional and network levels. The findings were largely consistent with our hypotheses. Globally, SSNHL exhibited a certain degree of compression in connectivity gradients, while the network gradient patterns remained consistent with the human connectome gradient framework. Regional functional connectivity gradients also showed disturbances, closely related to emotional and cognitive impairments.

Patients with SSNHL maintained consistency in the primary gradient from basic sensory cortices to higher-order cognitive cortices, showing no significant differences compared to the healthy control group. This consistency might be attributed to the acute nature of SSNHL, where the brief onset duration allows the body to compensate functionally for sensory loss in the early stages of the disease. However, gradient contraction was observed in both the second and third gradients. In the second gradient, subjects exhibited a unimodal area separation state. The increase in the gradient value of the SON and the decrease in the VIS gradient value led to a contraction of the functional gradient. This second gradient reflects the functional differentiation of the visual, somatosensory and auditory networks. The loss of hearing activates the brain’s compensation mechanisms, recruiting visual and somatosensory information to compensate for the reduced auditory stimuli. Previous studies have indicated that the absence of auditory input affects visual and somatosensory functions.^37^ This gradient contraction may enhance the information integration efficiency of the unimodal sensory network, thus serving a compensatory role.^36^ The third gradient also preserved the distribution state from unimodal areas to transmodal areas. Unlike the first gradient, the third gradient exhibited a pronounced state of contraction. Scholars have indicated that the third gradient represents anatomical centrality, a structural feature that is associated with systematic changes in the degree of cortical lamination differentiation.^38^However, this theory has not been further confirmed. Currently, there is no clear model for the distribution pattern of the third gradient. In a previous study on age-related hearing loss, the third gradient exhibited a state of separation in the transmodal areas, and the same state was observed in another study.^39^Further research and verification may be needed to understand the significance of the third gradient.

In the first gradient, a significant reduction in the functional connectivity gradient was observed in the left temporal pole region of SSNHL patients. The temporal pole, through its anatomical connections with the amygdala, choroid plexus and orbitofrontal cortex, influences cognitive functions such as emotion, attention, behaviour and memory.^40^ Located at the tip of the temporal lobe, the temporal pole plays a crucial role in various cognitive and emotional processes.^41^ It serves as an important hub connecting sensory networks with higher-order cognitive networks, integrating inputs from the sensory-motor-auditory network and relaying processed information to higher-order cognitive networks.^42^ During semantic tasks involving auditory stimuli, the left temporal pole is typically strongly activated. However, a reduction in the functional connectivity gradient of the temporal pole in SSNHL is understandable, as the absence of auditory stimuli prevents its activation.^41^ The stagnation of the temporal pole’s function disrupts the connection between unimodal and multimodal areas, akin to a bridge being severed, preventing effective communication between the two ends. This results in cognitive dysfunction in patients, as reflected in their neuropsychological test results. Notably, the gradient value of the temporal pole in SSNHL patients showed a significant positive correlation with their SDMT, AVLT and AVLT_delay scores, further indicating that the reduction in the temporal pole’s functional gradient is accompanied by a decline in cognitive and memory levels. Studies have demonstrated that patients with hearing loss exhibit a reduction in the grey matter volume of the temporal pole.^43^ Prolonged hearing loss also affects the functional connectivity of the temporal pole with adjacent tissues.^40^ Abnormal functioning of the temporal pole can lead to neurological diseases such as epilepsy and Alzheimer’s disease.^44^ It has been shown that patients with temporal lobe epilepsy experience improvement in symptoms of accelerated long-term forgetting and remote memory impairment after temporal pole resection.^45^ Additionally, a significant gradient decline was observed in the vPFC within the DMN. The vPFC, located at one end of the primary gradient, is responsible for emotional processing, social cognition and inhibitory control, as widely confirmed. It is also involved in speech comprehension and production.^46^ Furthermore, studies have shown that in noisy environments, the vPFC aids in speech perception and compensates for reduced activation in sensory areas.^47^ The loss of hearing increases cognitive load, especially in processing auditory information, potentially leading to functional remodelling and a decline in the functional connectivity gradient of the vPFC.^48^ Abnormal functioning of the vPFC can result in depressive symptoms, partly due to its impaired ability to regulate negative emotions.^49^ The vPFC is closely linked to the auditory system. It projects directly to the thalamic reticular nucleus and plays a key role in the inhibitory regulation of communication between the auditory thalamus and cortex. Effective output from the vPFC maintains the excitatory-inhibitory balance during auditory processing.^50^ Additionally, its decline in function affects reward processing, leading to anhedonia, a core symptom of depression.^51,52^ The decline in the vPFC’s functional connectivity gradient also affects its interaction with the limbic system, increasing the patient’s sensitivity to emotions and stress.^53^ This explains the significant negative correlation between the vPFC gradient value and HAMD scores in SSNHL patients in our experiment. The primary gradient represents the process of information integration from unimodal to higher-order cognitive areas. Although the absence of auditory input did not affect the global gradient pattern in SSNHL patients, the process of information integration and separation, aimed at compensating for the reduced auditory stimuli, led to functional remodelling due to cognitive load, resulting in abnormalities in the fine structure of function and consequently cognitive dysfunction in patients.

At the functional network level, SSNHL exhibited a distribution pattern in the primary gradient from basic sensory areas to higher-order cognitive areas. Consistent with the global gradient results, no significant inter-group differences were observed at the primary gradient level of the functional network. Conversely, in the second gradient, a significant reduction in the gradient value of the CON was observed. The CON, a group of regions involved in attentional cognitive control and emotional regulation, is responsible for language processing in the left hemisphere and decision-making and cognitive control in the right hemisphere.^54^ Patients with severe depression show reduced connectivity within the CON.^55^ It is believed that the decline in frontal lobe resting-state functional connectivity in patients with hearing loss is related to increased daily auditory effort rather than the hearing loss itself.^56^ Abnormal CON functional connectivity is observed in patients with hearing loss. Patients with unilateral hearing loss show reduced activation in the frontoparietal regions during visual working memory tasks, a result of the additional recruitment of auditory cognitive resources as a compensatory mechanism for reduced auditory input.^57^ Additionally, in the third gradient, subjects exhibited a distribution from the VIS/SOM to the CON/DMN, similar to the primary gradient. However, a significant increase in the gradient values of VIS and SOM was observed in SSNHL patients, likely a result of compensation by other sensory systems due to reduced auditory information. The significant reduction in the CON gradient value may be a consequence of limited cognitive resources compensating for the loss of sensory information. This change in gradient values also led to certain cognitive dysfunctions in patients. The cognitive process compensates for the degradation of auditory input. Reduced auditory acuity may reorganize the interactions between the sensory cortex and higher-order cortices. Therefore, cognitive and cross-modal processes are upregulated to compensate for hearing loss, consuming cognitive reserves. This may underlie the link between hearing loss and cognitive decline.

In this experiment, there were several limitations: (i) the small sample size of this study may limit the representativeness of the results, necessitating further validation with a larger sample size in subsequent research; (ii) the inclusion of SSNHL patients in this study encompassed both unilateral and bilateral sudden sensorineural hearing loss cases. Given the low incidence and specificity of bilateral SSNHL, the results of this study may exhibit some heterogeneity. Future studies should apply stricter criteria for participant selection; and (iii) given the limited research on the third gradient, further exploration and validation of the results and significance of the third gradient in this study are needed.

## Conclusion

This study, utilizing connectome gradients, reveals the network-level changes in SSNHL patients and their relationship with cognitive and emotional impairments. SSNHL exhibits abnormalities in functional connectivity gradients at global, regional and network levels. The absence of auditory input not only affects the normal functioning of other sensory systems but also consumes limited cognitive resources. These changes in functional gradients may be the underlying mechanism leading to cognitive and emotional disorders in patients. Our research provides new insights into understanding the cortical functional abnormalities and cognitive–emotional deficits in SSNHL.

## Supplementary Material

fcae317_Supplementary_Data

## Data Availability

All data will be available upon request from any qualified investigator.
