# Supplementary material for: The relationship between changes in functional connectivity gradients and cognitive–emotional disorders in sudden sensorineural hearing loss
Source: Brain Commun. 2024 Sep 19;6(5):fcae317. doi: 10.1093/braincomms/fcae317 (PMC11420982; doi:10.1093/braincomms/fcae317)
Supplement: fcae317_Supplementary_Data [file fcae317_supplementary_data.docx]

**Supplementary material**

Here are some general codes used in this study.

**step_0_convert_xcp_pconn_fisher_z.m**

clear;clc

xcp_outdir='H:\gradient_analysis\data\pconn_data';

conndir='H:\gradient_analysis\data\fisher_z_funcdata';

sublist=dir(fullfile(xcp_outdir,'sub*'));

if ~exist(conndir,'dir')

mkdir(conndir)

end

for i = 1:length(sublist)

subid=str2num(sublist(i).name(end-2:end));

schearfer_400 = cifti_read(fullfile(xcp_outdir,sprintf('sub-%03d',subid),sprintf('sub-%03d_task-rest_run-1_space-fsLR_atlas-Schaefer417_den-91k_bold.pconn.nii',subid)));

func_data = schearfer_400.cdata;

func_data=(0.5*log((1 + func_data)./(1 - func_data)));

for a = 1:length(func_data)

for b = 1:length(func_data)

if a == b

func_data(a,b) = 1;

end

end

end

subdir=fullfile(conndir,sprintf('sub-%03d',subid));

if ~exist(subdir,'dir')

mkdir(subdir);

end

save(fullfile(subdir,'func_scheafer400.mat'),'func_data');

end

**step_1_all_subjects_gradients.m**

clear;clc;

labeling = load('J:\tidu_lesson\8BNTG_day4\8BNTG_day4\scheafer_400_7network_order\label.mat');

root_dir = 'J:\tidu_result_step0';

out_path = 'J:\tidu_result_step1';

if ~exist(out_path,'dir')

mkdir(out_path);

end

data_all = dir([root_dir filesep 'sub-*']);

for sub = 1:numel(data_all)

sub_func = load([root_dir filesep data_all(sub).name filesep 'func_scheafer400.mat']);

gm = GradientMaps('kernel','normalizedAngle','approach','diffusionEmbedding');

gm = gm.fit(sub_func.func_data);

out_name = [out_path filesep data_all(sub).name];

if~exist(out_name,'dir')

mkdir(out_name);

end

save([out_name filesep 'gradient.mat'],'gm');

sub

end

**step_2_align_all_subjects.m**

clear;clc;

labeling = load('E:\tidu\8BNTG_day4\scheafer_400_7network_order\label.mat');

root_dir = 'E:\tidu\result\result_1';

out_path = 'E:\tidu\result\result_2';

if ~exist(out_path,'dir')

mkdir(out_path);

end

data_all = dir([root_dir filesep 'sub-*']);

n_sub= numel(data_all);

for sub = 1:n_sub

sub_func = load([root_dir filesep data_all(sub).name filesep 'gradient.mat']);

all_sub_gradients_explanation_ratio(:,sub)=sub_func.gm.lambda{1} ./ sum(sub_func.gm.lambda{1});

all_sub_gradients{sub} = sub_func.gm.gradients{1};

sub

end

[aligned, xfms] = procrustes_alignment(all_sub_gradients,'nIterations', 100);

for sub = 1:n_sub

aligned_gradient = aligned{sub};

aligned_gradient_all(:,:,sub)=aligned_gradient;

out_name = [out_path filesep data_all(sub).name];

if ~exist(out_name,'dir')

mkdir(out_name)

end

save([out_name, filesep, 'aligned_gradient.mat'],'aligned_gradient');

sub

end

labeling = load('E:\tidu\8BNTG_day4\scheafer_400_7network_order\label.mat');

labeling = labeling.schaefer_400;

[surf_lh, surf_rh] = load_conte69();

plot_hemispheres([aligned{10}(:,1),aligned{2}(:,1),aligned{7}(:,1),aligned{30}(:,1)], ...

{surf_lh,surf_rh}, 'parcellation', labeling, ...

'labeltext',{'aligned sub 10','aligned sub 25','aligned sub 35','aligned sub 50'});

group_dir = 'E:\tidu\result\group';

if ~exist(group_dir,'dir')

mkdir(group_dir);

end

group1_gradient= mean(aligned_gradient_all(:,:,1:30),3);

group2_gradient= mean(aligned_gradient_all(:,:,31:end),3);

h=plot_hemispheres([group1_gradient(:,3), group2_gradient(:,3)],{surf_lh,surf_rh}, ...

'parcellation', labeling,'labeltext',{'HC','PC'});

colormap(h.handles.figure,jet(401));

save([group_dir '\group1_mean_gradient.mat'],'group1_gradient');

save([group_dir '\group2_mean_gradient.mat'],'group2_gradient');

save([group_dir '\all_subjects_aligned_gradient.mat'],'aligned_gradient_all');

figure;h1=histfit(group1_gradient(:,1),[],'kernel');hold on; h2=histfit(group2_gradient(:,1),[],'kernel');

h1(1).FaceAlpha = 0.2;h2(1).FaceAlpha = 0.2;h1(2).Color = [.9 .4 .15];h2(2).Color = [.2 .2 .9];

figure;h1=histfit(group1_gradient(:,2),[],'kernel');hold on; h2=histfit(group2_gradient(:,2),[],'kernel');

h1(1).FaceAlpha = 0.2;h2(1).FaceAlpha = 0.2;h1(2).Color = [.9 .4 .15];h2(2).Color = [.2 .2 .9];

figure;h1=histfit(group1_gradient(:,3),[],'kernel');hold on; h2=histfit(group2_gradient(:,3),[],'kernel');

h1(1).FaceAlpha = 0.2;h2(1).FaceAlpha = 0.2;h1(2).Color = [.9 .4 .15];h2(2).Color = [.2 .2 .9];

emb_range = zeros(n_sub,3);

for i = 1:3

for j = 1:n_sub

emb_range(j,i) = max(aligned_gradient_all(:,i,j)) - min(aligned_gradient_all(:,i,j));

end

end

save([group_dir,'/emb_range.mat'],'emb_range');

emb_std = zeros(n_sub,3);

for i = 1:3

for j = 1:n_sub

emb_std(j,i) = std(aligned_gradient_all(:,i,j));

end

end

save([group_dir,'/emb_std.mat'],'emb_std');

**step_3_group_stats.m**

clear;clc;

all_sub_data = load('E:\tidu\result\result_g\all_subjects_aligned_gradient.mat');

all_sub_data = all_sub_data.aligned_gradient_all;

HC = 1:30;

SSNHL = 31:60;

for n_dim = 1:3

HC_all = squeeze(all_sub_data(:,n_dim,HC))';

SSNHL_all = squeeze(all_sub_data(:,n_dim,SSNHL))';

for n_parcle = 1:400

[~,p_tmp,~,stat_T]=ttest2(HC_all(:,n_parcle),SSNHL_all(:,n_parcle));

t(n_parcle)=stat_T.tstat;

p(n_parcle)=p_tmp;

end

[p_mask,~,~,adj_p] = fdr_bh(p);

all_t(n_dim,:) = t;

all_p(n_dim,:) = p;

all_p_fdr_mask(n_dim,:) = p_mask;

all_t_fdr_correct(n_dim,:) = t.*p_mask;

all_t_uncorrect(n_dim,:)=t.*double(p<0.05);

all_p_adj(n_dim,:) = adj_p;

end

labeling = load('E:\tidu\8BNTG_day4\scheafer_400_7network_order\label.mat');

labeling = labeling.schaefer_400;

[surf_lh, surf_rh] = load_conte69();

plot_hemispheres([all_t_fdr_correct(1,:)' all_t_fdr_correct(2,:)' all_t_fdr_correct(3,:)'],{surf_lh,surf_rh},'parcellation',labeling, ...

'labeltext', {'gradient1','gradient2','gradient3'});

%% group stats on range

clear;clc;

all_sub_data = load('J:\group_result\emb_range.mat');

emb_range = all_sub_data.emb_range;

HC = 1:30;

SSNHL = 31:60;

% gradient range

range_stat = zeros(3,4);

n_mTBI = length(find(HC));

n_HC = length(find(SSNHL));

for i = 1:3

range_stat(i,1) = mean(emb_range(SSNHL,i));

range_stat(i,2) = std(emb_range(SSNHL,i));

range_stat(i,3) = mean(emb_range(HC,i));

range_stat(i,4) = std(emb_range(HC,i));

[~,p,~,stat_T] = ttest2(emb_range(HC,i),emb_range(SSNHL,i));

p_all_range(i)=p;

t_all_range(i)=stat_T.tstat(1);

end

disp(range_stat)

disp(p_all_range)

disp(t_all_range)

%% group stats on variance

clear;clc;

all_sub_data = load('J:\group_result\emb_std.mat');

emb_std = all_sub_data.emb_std;%sub*dim

HC = 1:30;

SSNHL = 31:60;

% gradient range

std_stat = zeros(3,4);

n_mTBI = length(find(HC));

n_HC = length(find(SSNHL));

for i = 1:3

std_stat(i,1) = mean(emb_std(SSNHL,i));

std_stat(i,2) = std(emb_std(SSNHL,i));

std_stat(i,3) = mean(emb_std(HC,i));

std_stat(i,4) = std(emb_std(HC,i));

[~,p,~,stat_T] = ttest2(emb_std(HC,i),emb_std(SSNHL,i));

p_all_dim(i)=p;

t_all_dim(i)=stat_T.tstat(1);

end

disp(std_stat)

disp(p_all_dim)

disp(t_all_dim)
